# Supplementary material for: Reliable and efficient transcranial magnetic stimulation–electroencephalography (TMS–EEG) using ultra-thin active electrodes
Source: Commun Eng. 2025 Nov 28;4:206. doi: 10.1038/s44172-025-00538-8 (PMC12663230; doi:10.1038/s44172-025-00538-8)
Supplement: Supplementary file 2 — Supplementary Information [file 44172_2025_538_MOESM2_ESM.pdf]

Supplementary Material

Reliable and Efficient Transcranial Magnetic Stimulation–Electroencephalography (TMS–EEG) Using Ultra-Thin Active Electrodes

Johannes Grünwald, Leonhard Schreiner, Sebastian Sieghartsleitner,

Alexandru Buzamat, Giovanni Lombardi, Antonio Calzone, Marco Fummo,

Slobodan Tanackovic, Marian-Silviu Poboroniuc, Rossella Spataro, Agnese Zazio,

Marta Bortoletto, and Christoph Guger

Supplementary Discussion 1 – TEP Time Courses

Figure S1 corresponds to Figure 1 in the main manuscript and presents a topographical overview of TEPs using the passive instead of the active reference electrode.

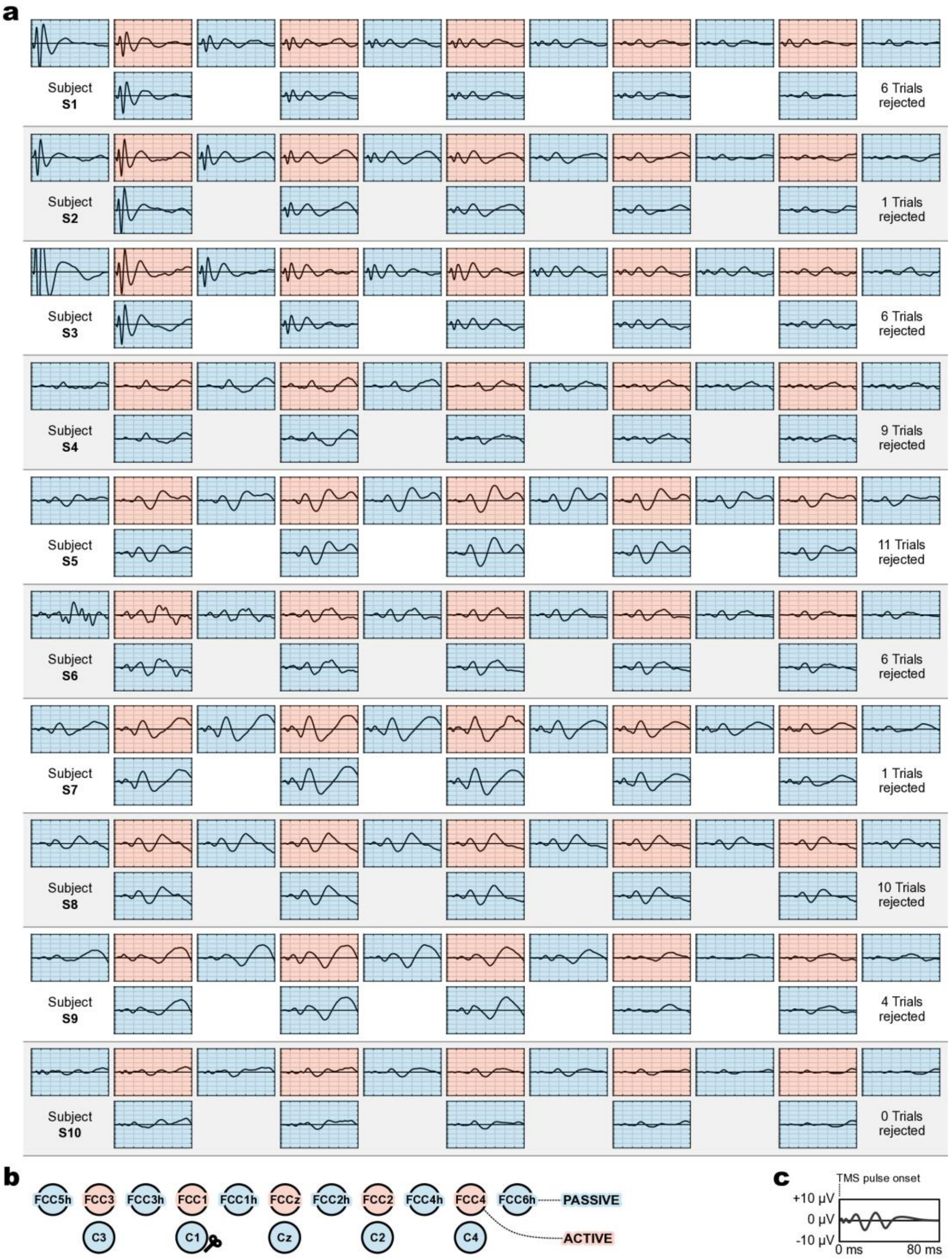

Figure S1: Topographical distribution of TMS-evoked potentials (TEPs) across subjects. a: Subject-specific visualization (S1-S10). b: Electrode montage. c: Axes. Light blue and red shading indicate passive and active electrode type, respectively. Coil location was above C1 as indicated.

Supplementary Discussion 2 – Decay Artifact Removal

Figure S2 corresponds to Figure 2 in the main manuscript but uses passive referencing instead of active referencing. We observed that the decay artifacts in the passive electrodes were substantially smaller, due to the fact that also the reference electrode was passive. Interestingly, the very early responses in S3 appeared less prominent with passive referencing. Instead, these components seemed more spatially confined to the stimulation site, as shown in Figure S1 compared to Figure 1 in the main manuscript. A possible explanation is that the active reference electrode introduced components of these very early responses into the signal, which caused their overall visibility across all electrodes.

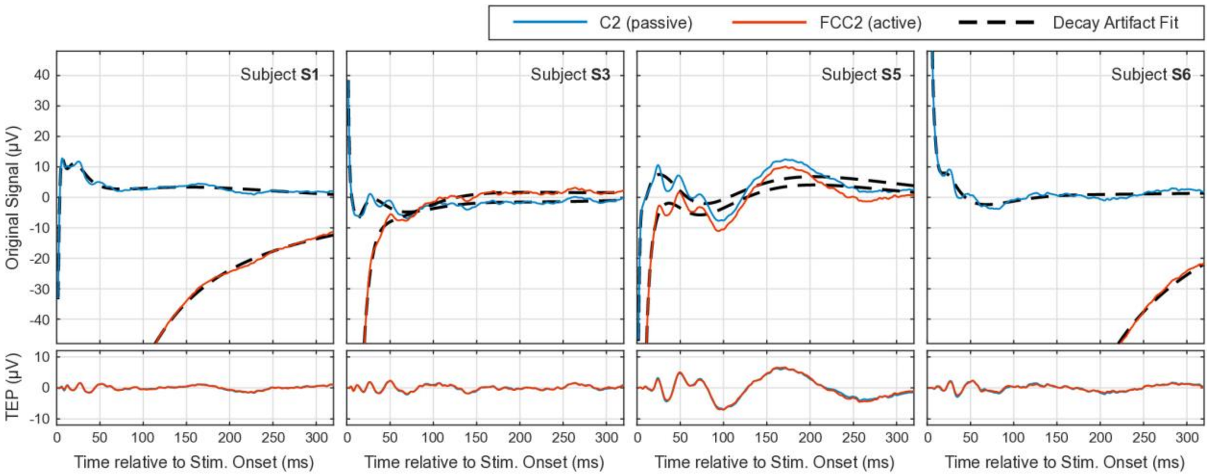

Figure S2: Examples of decay artifact removal performance. Upper plots: Original epoched and averaged signals with the decay artifact fit. Lower plots: Resulting TMS-evoked potentials (TEPs) after artifact removal. Note the identical amplitude scale across all plots.

Supplementary Discussion 3 – Signal Consistency

Figure S3 corresponds to Figure 3 in the main manuscript, but uses passive instead of active referencing. In Figure S3a, we analyzed all available neighboring electrode pairs across both electrode types, resulting in 15 pairs per subject and a total of N = 150 pairs, each separated by an approximate distance of Δ ≈ 2.1 cm. For early TEPs (15–80 ms post-stimulus), we observed a median concordance correlation coefficient (CCC) of 0.96 with an interquartile range (IQR) of [0.91, 0.98]. For late TEPs (80–350 ms), the median CCC was 0.95 [0.89, 0.98]. In Figure S3b, we assessed signal consistency within passive electrodes by analyzing diagonal neighbors (N = 100, Δ ≈ 3.0 cm), yielding a CCC of 0.94 [0.88, 0.97] for early and 0.94 [0.90, 0.97] for late TEPs. Figure S3c shows the analysis of horizontal neighbors within passive electrodes (N = 50, Δ ≈ 4.2 cm), resulting in a CCC of 0.87 [0.72, 0.93] for early TEPs and 0.89 [0.82, 0.94] for late TEPs.

Finally, due to the topography of the active electrode layout, only horizontal neighbor comparisons were feasible (Figure S3d). Here, the CCC was 0.88 [0.76, 0.93] for early TEPs and 0.87 [0.77, 0.93] for late TEPs.

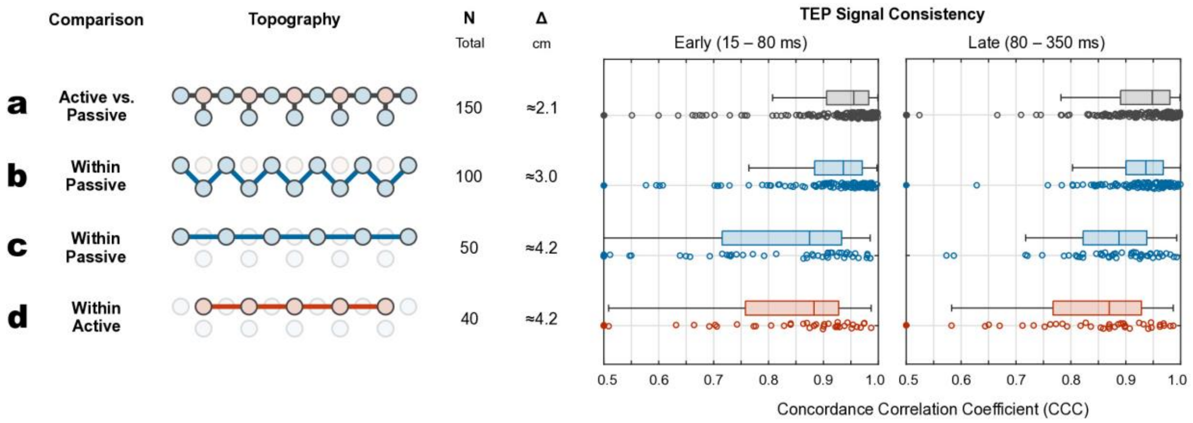

Figure S3: Signal consistency analysis across different electrode configurations. a: Active vs. passive electrodes. b: Within passive electrodes – diagonal pairs. c: Within passive electrodes – horizontal pairs. d: Within active electrodes – horizontal pairs. Light blue and red shading indicate passive and active electrode type, respectively. N: total number of electrode pairs across all subjects. Δ: approximate distance between electrode pairs.

Supplementary Discussion 4 – Amplitude Variability

We analyzed TEP amplitudes (root mean square, RMS) using passive instead of active referencing for early (15–80 ms) and late (80–350 ms) components using linear mixed-effects models. In both time windows, TEP amplitude decreased significantly with increasing distance from the stimulation site (early: $\beta = -0.126$, $p < 0.001$; late: $\beta = -0.080$, $p < 0.001$). Electrode type (active vs. passive) had no significant effect (early: $\beta = -0.036$, $p = 0.62$; late: $\beta = -0.093$, $p = 0.09$). The models revealed substantial between-subject variability in overall TEP amplitude (intercept standard deviation (SD): 0.85 early, 1.21 late), and in the effects of distance (SD: 0.06 early, 0.06 late) and electrode type (SD: 0.06 early, 0.05 late). Residual variability was relatively low (SD: 0.36 early, 0.27 late), indicating that the inclusion of random slopes captured meaningful inter-individual differences in how electrode type and distance influenced TEPs.

Figure S4, corresponding to Figure 4 in the main manuscript, provides a qualitative visualization of the TEP amplitudes across the anterior row of the electrode montage. A clear amplitude gradient with increasing distance from the stimulation site (C1) is visible for both early and late components. For clarity, we normalized amplitudes for each subject by the average RMS across all channels.

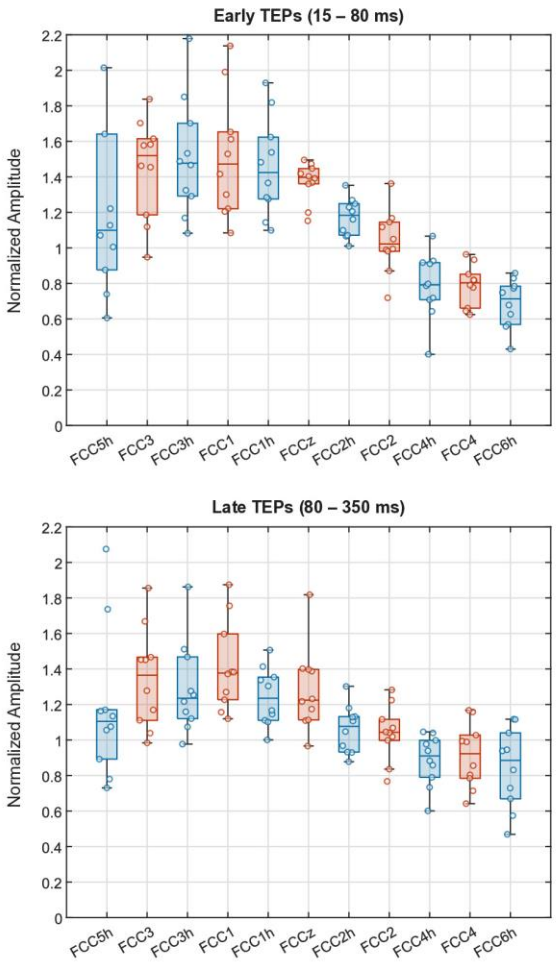

Figure S4: Lateral progression of normalized TMS-evoked potential (TEP) amplitudes across the anterior electrode row. Values are shown from leftmost (FCC5h) to rightmost (FCC6h) electrode. Blue: passive electrodes; red: active electrodes.

Supplementary Discussion 5 – Convergence of Averages

Figure S5, corresponding to Figure 5 in the main manuscript, shows the convergence of averages for early and late TEPs toward the full-trial average across all available trials, using passive instead of active referencing. Active and passive electrodes exhibit nearly identical convergence behavior. Early TEPs converge more rapidly, reaching a median CCC of 0.8 after 20 trials, whereas approximately 40 trials are needed for late TEPs. Likewise, a median CCC of 0.9 is achieved after 30 trials for early TEPs, but it takes around 60 trials to reach the same level for late TEPs.

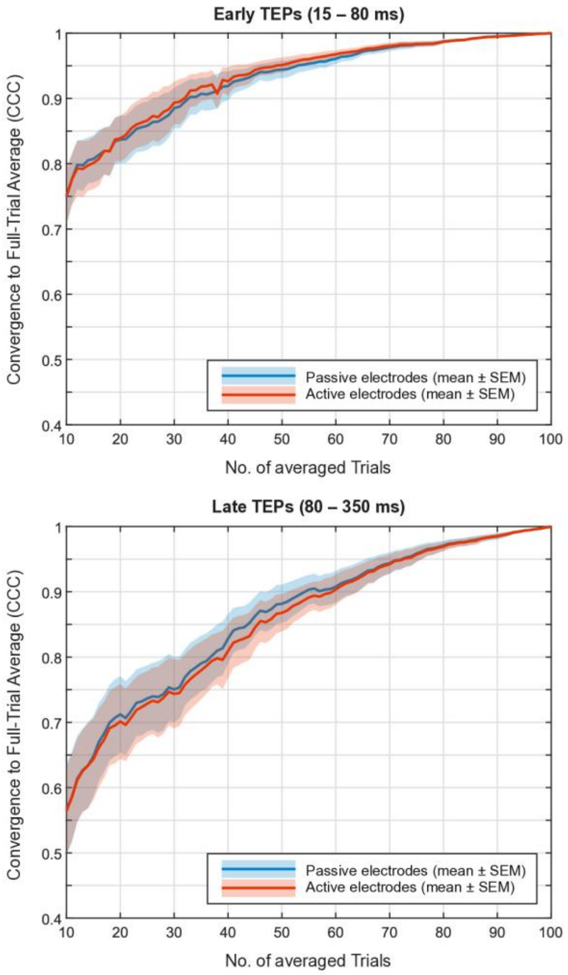

Figure S5: Convergence to the full-trial TMS-evoked potential (TEP) average. The solid line represents the mean, and the shaded area indicates the standard error of the mean (SEM). Passive and active electrodes are shown in blue and red, respectively.
